# Evaluation of the Possible Transmission of BSE and Scrapie to Gilthead Sea Bream (*Sparus aurata*)

**DOI:** 10.1371/journal.pone.0006175

**Published:** 2009-07-28

**Authors:** Evgenia Salta, Cynthia Panagiotidis, Konstantinos Teliousis, Spyros Petrakis, Eleftherios Eleftheriadis, Fotis Arapoglou, Nikolaos Grigoriadis, Anna Nicolaou, Eleni Kaldrymidou, Grigorios Krey, Theodoros Sklaviadis

**Affiliations:** 1 Department of Pharmacology, Aristotle University of Thessaloniki, Thessaloniki, Greece; 2 Centre for Research and Technology-Hellas, Institute of Agrobiotechnology, Thessaloniki, Greece; 3 Laboratory of Pathology, School of Veterinary Medicine, Aristotle University of Thessaloniki, Thessaloniki, Greece; 4 Max Delbruck Center for Molecular Medicine, Department of Neuroproteomics, Berlin-Buch, Germany; 5 National Agricultural Research Foundation, Fisheries Research Institute, Nea Peramos, Greece; 6 B' Department of Neurology, AHEPA University Hospital, Aristotle University of Thessaloniki, Thessaloniki, Greece; 7 Department of Business Administration, University of Macedonia, Thessaloniki, Greece; Université de Toulouse, France

## Abstract

In transmissible spongiform encephalopathies (TSEs), a group of fatal neurodegenerative disorders affecting many species, the key event in disease pathogenesis is the accumulation of an abnormal conformational isoform (PrP^Sc^) of the host-encoded cellular prion protein (PrP^C^). While the precise mechanism of the PrP^C^ to PrP^Sc^ conversion is not understood, it is clear that host PrP^C^ expression is a prerequisite for effective infectious prion propagation. Although there have been many studies on TSEs in mammalian species, little is known about TSE pathogenesis in fish. Here we show that while gilthead sea bream (Sparus aurata) orally challenged with brain homogenates prepared either from a BSE infected cow or from scrapie infected sheep developed no clinical prion disease, the brains of TSE-fed fish sampled two years after challenge did show signs of neurodegeneration and accumulation of deposits that reacted positively with antibodies raised against sea bream PrP. The control groups, fed with brains from uninfected animals, showed no such signs. Remarkably, the deposits developed much more rapidly and extensively in fish inoculated with BSE-infected material than in the ones challenged with the scrapie-infected brain homogenate, with numerous deposits being proteinase K-resistant. These plaque-like aggregates exhibited congophilia and birefringence in polarized light, consistent with an amyloid-like component. The neurodegeneration and abnormal deposition in the brains of fish challenged with prion, especially BSE, raises concerns about the potential risk to public health. As fish aquaculture is an economically important industry providing high protein nutrition for humans and other mammalian species, the prospect of farmed fish being contaminated with infectious mammalian PrP^Sc^, or of a prion disease developing in farmed fish is alarming and requires further evaluation.

## Introduction

Transmissible spongiform encephalopathies or prion diseases are a group of fatal neurodegenerative disorders, including Creutzfeldt-Jakob disease (CJD), Fatal Familial Insomnia (FFI) and Gerstmann-Sträussler-Scheinker disease (GSS) in humans, scrapie in sheep and goats and bovine spongiform encephalopathy (BSE) in cattle [1].

The transmission of clinical prion diseases is limited by the so-called “species barrier” to conversion of endogenous host prion protein (PrP^C^) to its abnormal, partially proteinase K-resistant conformational isoform, PrP^Sc^. When high enough, this “barrier” can greatly impair or prevent potential interspecies transmissions, even under optimal conditions of dose and infection route. However, evidence of TSE replication without accompanying symptoms of clinical disease has prompted debate on the existence of asymptomatic infected individuals in an exposed population [2], [3].

The identification of apparent PrP orthologues in lower vertebrates, including fish [4]–[16], raises the question of their susceptibility to prion diseases. While fish PrP-like sequences do not share high homology with their mammalian relatives (Table S1), they do contain several strongly conserved prion protein structural motifs [17]. Although mammalian to fish TSE transmission is considered unlikely [18], it is not certain that the species barrier would be high enough to prevent TSE transmission to fish.

The BSE epidemic has been linked to TSE-infected cattle feed [19] and the recognition of BSE in domestic cattle inevitably raised concerns about the potential risk to other ruminant and non-ruminant livestock [20]. The European Commission's TSE risk-reducing measures include a total EU-wide ban on the use of all processed animal protein in livestock and aquaculture feeds. Any consideration of lifting this ban requires a scientific assessment of the TSE transmission risk through fishmeal. Another issue to be addressed is the rising concern that pigs, poultry or fish bred for human consumption and inadvertently fed with TSE-contaminated feed could eventually either develop clinical TSE or serve as reservoirs of infectivity without ever displaying clinical disease themselves. Such an assessment should consider the risk from TSE-contaminated feed being fed to farmed fish [18], [21]. In aquaculture, a rapidly growing industry of economic importance in several EU countries, the farmed fish receive commercial feed containing 40–55% protein during the 12–20 months they generally spend in aquaculture facilities. Although remote, the possibility that some of this feed might be contaminated with mammalian prion cannot be excluded.

In the present work, we evaluated the potential transmission of TSEs to gilthead sea bream, a commercially important fish species. After force-feeding with multiple doses of brain homogenate prepared from either healthy or naturally BSE- or scrapie-infected cow or sheep, the fish were monitored for 2 years for evidence of disease development by clinical, histopathological and immunohistochemical criteria. None of the fish examined, showed symptoms of clinical disease. However, signs of neurodegeneration were often present and abnormal deposition was detected in the brains of both the scrapie-challenged and the BSE-challenged fish by 24 months post inoculation.

## Results and Discussion

To evaluate the clinical state of the fish, we monitored control and TSE–challenged populations on a daily basis. Since locomotor deficits are often a major feature of the clinical presentation of prion diseases in a variety of hosts, we used the swimming behavior of the challenged fish as an indicator of their general activity and exploratory behavior. No clinical symptoms, including erratic swimming or behavioral abnormalities, were observed in any of the groups monitored. Although unusual in prion disease, a similar absence of clinical symptoms upon interspecies challenge has been reported for both the first passage of sheep scrapie and hamster prion transmission to mice [2]. In these cases, subsequent passage of brain material from the challenged individuals to additional mice did produce clinical disease, thereby demonstrating that asymptomatic animals can harbour high levels of infectious prions in their brains. Additionally, it is important to note that while certain experimentally or virally induced neurodegenerative effects do modify swimming parameters in fish, such as the swimming distance and orientation, the mean velocity, the turning angle and the equilibrium [22]–[24], this is not always the case. For instance, while both sea bass and sea bream can be infected with nodavirus, a naturally occurring piscine virus that causes brain lesions in both species, sea bream, in contrast to sea bass, show no clinical symptoms of disease [25], [26].

To facilitate our evaluation we generated polyclonal antibodies against four different fish PrPs. The specificity of each antiserum was confirmed by both western blot (Fig. S1A) and immunohistochemistry (IHC) (Fig. S2A–C) with normal sea bream brain. Furthermore, anti-mammalian PrP antibodies (6H4, 12F10) did not stain sea bream brain, nor did our anti-fish PrP antisera recognize mammalian PrP^Sc^ (Fig. S1B). Moreover, absorption of our SaurPrP1 (*Sparus aurata* PrP1) antisera with recombinant sea bream PrP-1 protein [6], against which it was raised, resulted in a complete loss of its specific immunostaining in control fish (Fig. S2D).

**Figure 1:**
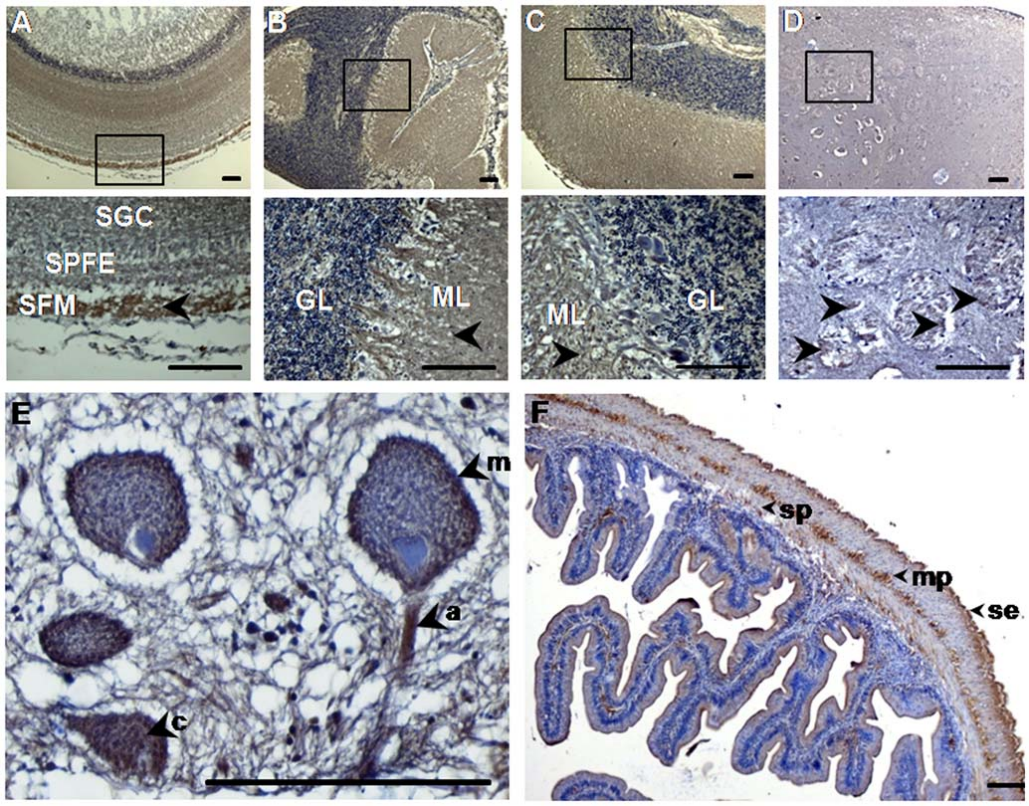
Normal PrP^C^ distribution in CNS and peripheral tissues. Sagittal, 4 µm-thick brain and intestine sections from control fish were treated with SaurPrP1 (1∶2000 and 1∶250, respectively) and normal endogenous PrP labeling in different anatomical regions was examined. A, Optic tectum; B, Valvula cerebelli; C, Corpus cerebelli; D, Nerve fibers in diencephalon; E, Neurons in brainstem; F, PrP-immunoreactive areas in the intestine. Rectangles indicate areas of magnification shown in the panel directly below. Arrowheads show positively stained regions. SGC, striatum griseum centrale; SPFE, striatum plexiforme et fibrosum externum; SFM, striatum fibrosum marginale; ML, molecular layer; GL, granular layer, m, plasma membrane; a, axon; c, cytoplasm. se, serosa; mp, myenteric plexus; sp, submucous plexus. Scale bars, 100 µm.

Initially, in order to determine the distribution of normal endogenous PrP in the central nervous system of gilthead sea bream, we used our polyclonal antisera to perform a detailed immunohistochemical evaluation on brain sections from control fish. Regions displaying abundant PrP included the optic tectum (Fig. 1A), valvula cerebelli (Fig. 1B) and corpus cerebelli (Fig. 1C), while strong PrP-immunopositivity was generally observed in the nerve fibers (Fig. 1D). The most prominently stained regions of the optic tectum, homologue to the superior colliculus in mammals [22], were striatum fibrosum marginale (Fig. 1A and Fig. S2A, B) and striatum fibrosum profundum (Fig. S2A, B). Less intense labeling was observed in the striatum griseum centrale and striatum plexiform fibrosum externum layers of the optic tectum (Fig. 1A and Fig. S2A, B). Cerebellar PrP-immunopositivity was detected mainly in the molecular layer, between Purkinje cell dendrites, and in the granular layer, in the matrix surrounding the granule cells (Fig. 1C). The valvula cerebelli, a rostral protrusion of the cerebellum in the midbrain ventricle that has no counterpart in mammals, showed significant PrP-immunopositivity, similar to that observed in the molecular and granular layers of cerebellum (Fig. 1B and Fig. S2A, B). In cerebral regions, including thalamus, medulla oblongata, diencephalon and the lateral telencephalic pallium, proposed to be a homologue of the mammalian hippocampus [22], we observed intense labeling of fiber bundles (Fig. 1D), consisting primarily of dendritic and axonal prolongations in the neuropil. The same general PrP^C^ expression pattern was observed in challenged and control populations, with no variation detected over time. The remarkable similarity of the overall immunolabeling pattern obtained with our SaurPrP1 antiserum in sea bream brain to the PrP-immunostaining profile in the mammalian brain [27], [28] provided further assurance of the specificity of our antibody for piscine PrP.

At the intracellular level, staining outlining the neuronal body was present in most of the neuronal populations observed, e.g. in the large neurons of the brainstem (Figure 1E). Axons displayed intense staining, while diffuse staining was observed inside some of these neuronal somata, suggesting a degree of PrP-immunopositivity within cell departments, e.g. the Golgi complex (Fig. 1E). These findings suggest that the intracellular localization of PrP in fish brain is comparable to the neuronal intracellular localization of mammalian PrP [27], [29].

To test for pathology in selected peripheral tissues, we examined intestines and spleens from TSE-challenged fish, sampled at different timepoints. No lesions or any other abnormalities were revealed in comparison to the control individuals. Intestinal PrP-immunoreactivity was evident in the serosa, myenteric plexus and submucous plexus (Fig. 1F). At all timepoints, PrP-immunolabeling in spleen and intestinal tissue was similar in both TSE-challenged and control fish and revealed no PK-resistance and no lesions or any other abnormalities.

**Figure 2:**
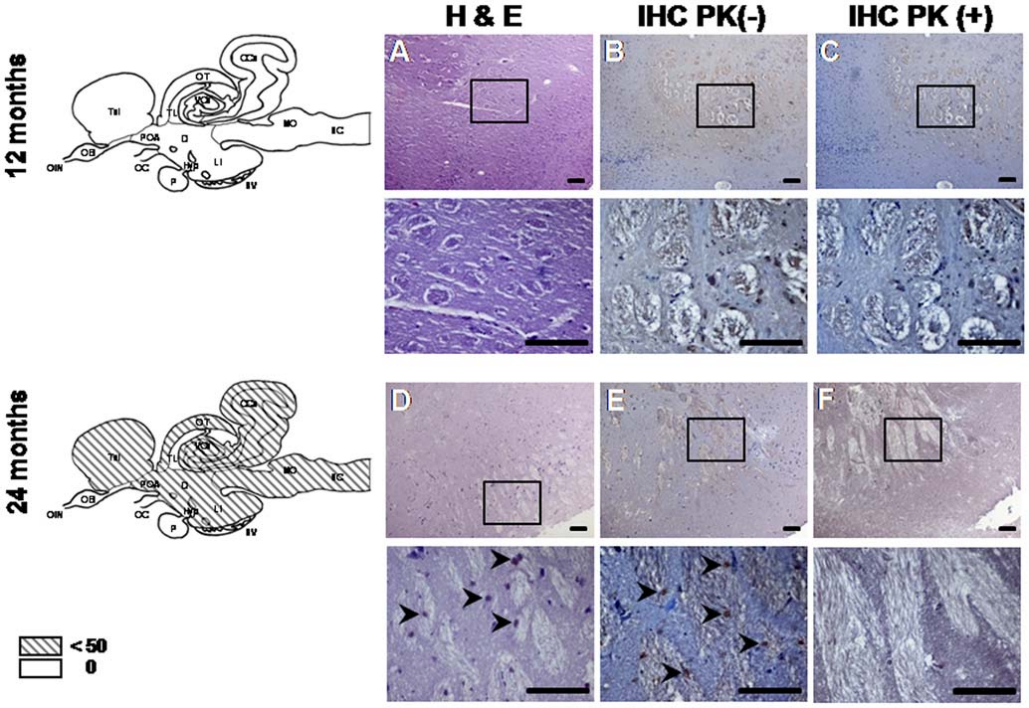
Progression of abnormal deposition in 2 scrapie-challenged fish. Sagittal brain sections from scrapie-challenged fish at 12 and 24 months p.i. were stained with H&E (A, D), or treated with SaurPrP1 (1∶2000) without PK-digestion (B, E) and with PK-digestion (C, F). Images show diencephalon. The mean number of deposits (per section of fish containing deposits) observed in different brain regions without PK-treatment is indicated by the fill-type in the schematic drawings at the far left. CCe, corpus cerebelli; Di, diencephalon; Hyp, hypothalamus; LI, lobi inferioris; MO, medulla oblongata; OB, olfactory bulb; OC, optic chiasm; OlN, olfactory nerve; OT, optic tectum; P, pituitary; POA, preoptic area; SC, spinal cord; Tel, telencephalon; TL, torus longitudinalis; VCe, valvula cerebelli. The following areas were not examined: OB; OIN; OC; P. Rectangles indicate areas of magnification shown in the panels directly below. Arrowheads indicate the abnormal aggregates. Scale bars, 100 µm.

Detailed examination of brain sections revealed no histopathological evidence of disease in scrapie–challenged sea bream through 18 months post inoculation (p.i.). At 24 months, however, 2 out of 5 fish showed limited abnormal, PrP-immunoreactive, PK-sensitive, extracellular deposits (Table S3B) in the neuropil of brainstem, diencephalon, corpus cerebelli, valvula cerebelli, optic tectum and telencephalon (Fig. 2). Whilst the number of animals where plaques were found was too small to reach statistical significance, based on the high likelihood that the fish examined developed no plaques, we believe that the observation of aggregates in 2 out of 5 fish at the final time point could be considered as an important event of qualitative (and not quantitative) value (Text S1). No lesions were detected in the control fish force-fed normal sheep brain homogenate (Fig. S3 and Table S3B).

**Figure 3:**
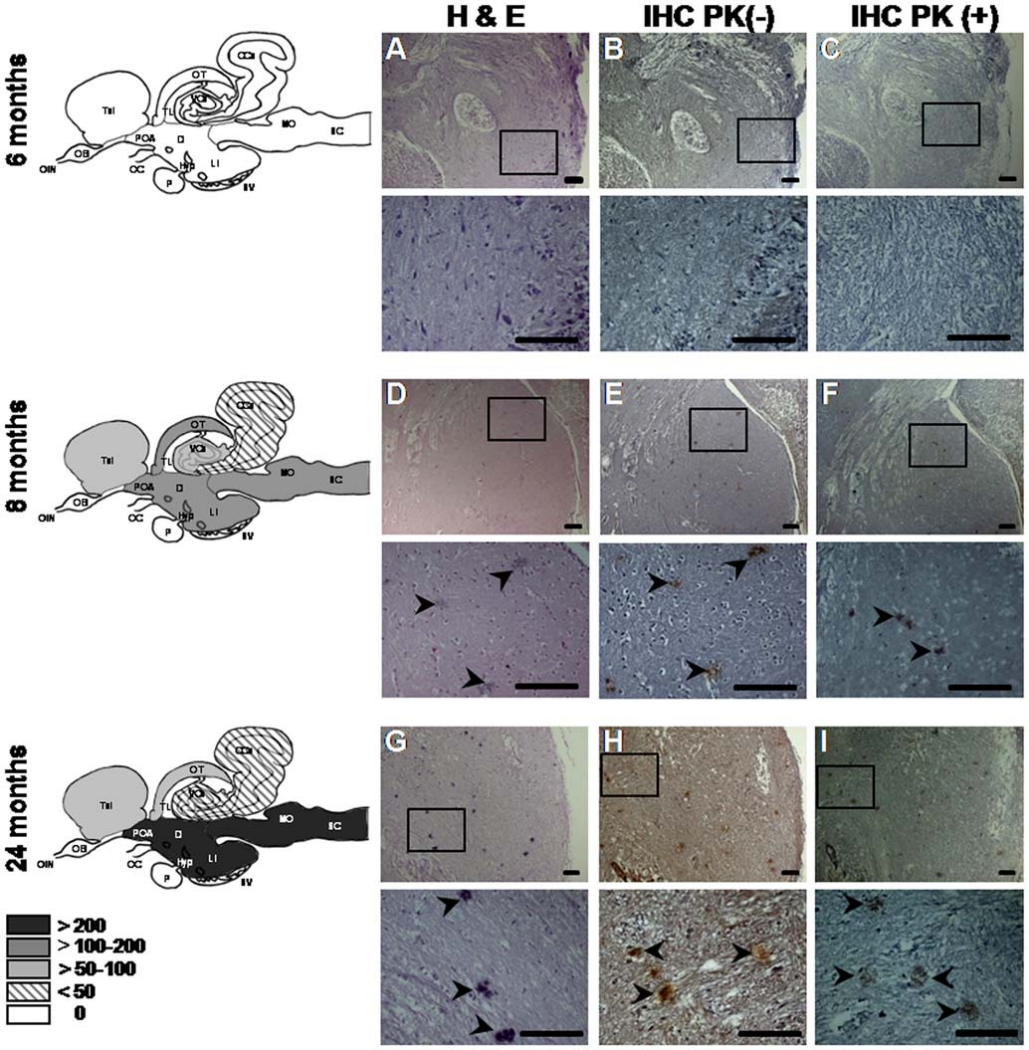
Progression of abnormal deposition in BSE-challenged fish. Sagittal brain sections from BSE-challenged fish taken at the indicated times p.i. were stained with H&E (A, D, G), or immunolabeled with SaurPrP1 (1∶2000) without PK-digestion (B, E, H) and with PK-digestion (C, F, I). Images show diencephalon. The mean number of deposits (per section of fish containing deposits) observed in different brain regions without PK-treatment is indicated by the fill-type in the schematic drawings at the far left. Abbreviations as in Figure 2. The following areas were not examined: OB; OIN; OC; P. Rectangles indicate areas of magnification shown in the panels directly below. Arrowheads indicate the abnormal aggregates. Scale bars, 100 µm.

Plaque-like deposits were also observed in the brains of the BSE-challenged fish, beginning at earlier timepoints. Initially, at 8 months p.i., the majority of these aggregates were localized in brainstem, less in diencephalon and optic tectum, and even fewer in valvula cerebelli, cerebellum and telencephalon (Figure 3D–F). Just 10% of the deposits were PK-resistant and these had a mean diameter of 5 µm. Subsequently, we observed a general progression in their distribution, size, PK-resistance and morphological features. The incidence of the abnormal deposition was higher in fish sacrificed at earlier time points than at intermediate time points. However, the highest levels were measured at later time points, evocative of the phenomenon described in other prion cross-species transmission studies as an “eclipse” period [30].

**Figure 4:**
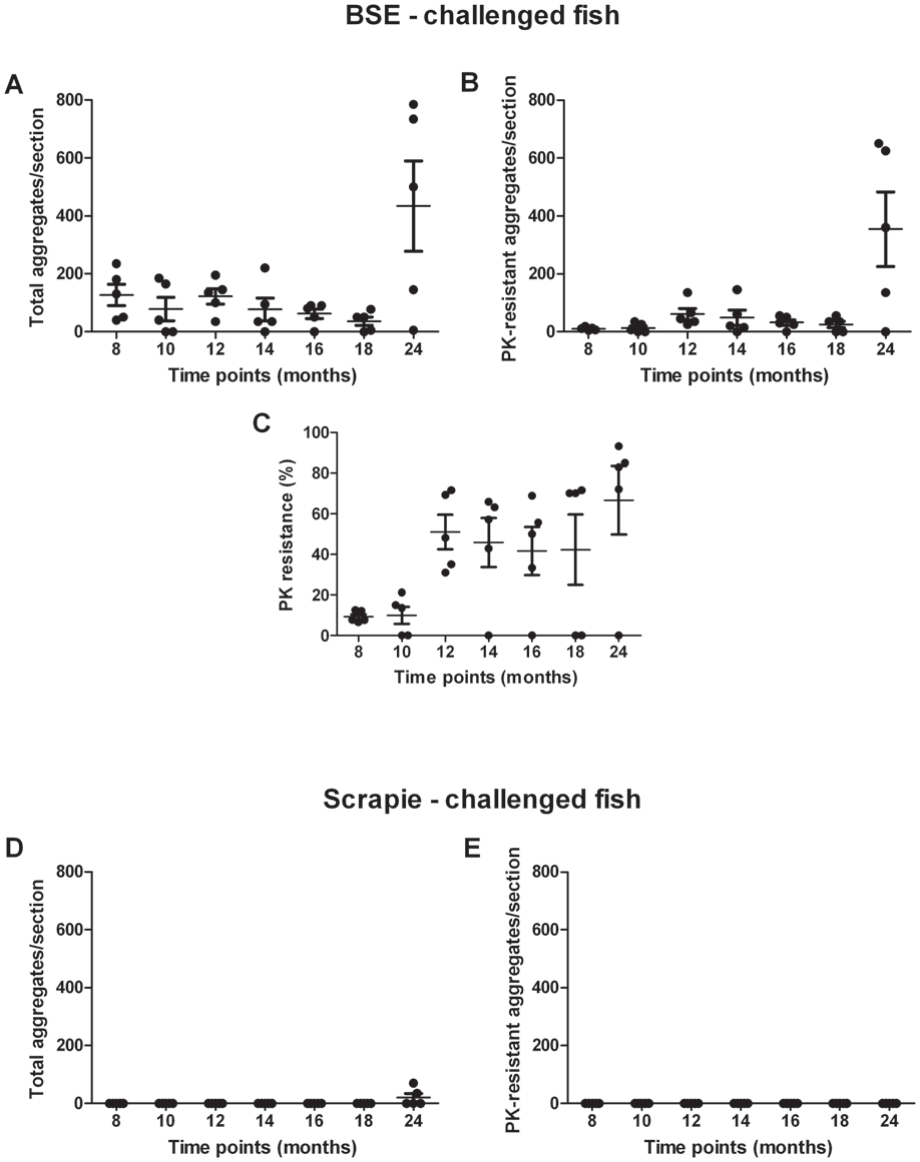
Abnormal deposits in the brains of the BSE- and the scrapie-challenged fish with reference to time. Each dot corresponds to the number of aggregates observed per brain section in each individual before PK-treatment (A, D) and after PK digestion (B, E), or to the percentage of PK-resistant aggregates in each BSE-challenged fish (C). Bars indicate the means and the standard error means (SEMs).

**Figure 5:**
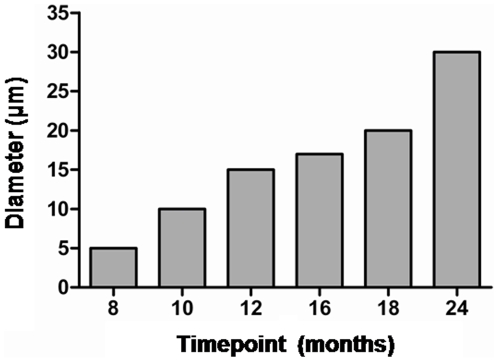
Progressive increase in the size of proteinase K resistant deposits in BSE-challenged fish. The mean diameter of the immunohistochemically detected (SaurPrP1) deposits after proteinase K–digestion is given with reference to time p.i..

Further analysis of the spatial and temporal progression revealed that with increasing time p.i., the deposition became more prominent in rostral brain regions, although caudal regions continued to be affected. By 24 months p.i., deposition in the brains of the BSE-challenged sea bream presented a striking picture, in which three out of five fish showed 500–800 deposits each, 70–85% of which were PK-resistant with a mean diameter of 30 µm. With regard to the remaining two fish, one displayed approximately 150 deposits, 93% of which were PK-resistant and the other showed only limited signs of abnormal aggregation. While deposits continued to be distributed throughout the brain at 24 months p.i., in the three highly affected fish the greatest increases in deposit numbers occurred in brainstem and diencephalon. The progression of the abnormal deposition is apparent in Fig. 3. and summarized in Figs. 4 and 5 and Tables S3A and S4.

In contrast to the BSE-challenged fish, no aggregates were detected at any time in the brains of the control fish fed with normal bovine brain homogenate (Fig. S4 and Table S3A). Notably, none of the brain tissues positive for abnormal deposition showed evidence of neuronal body degeneration. Finally, no residual mammalian PrP^Sc^ was detected using 12F10 and 6H4 monoclonal antibodies (data not shown). Overall, these data suggest that while both TSE strains resulted in similar abnormal brain pathology, the brains of BSE-challenged individuals were more rapidly and severely affected than those of scrapie-challenged fish. BSE, known to be a zoonotic TSE, may represent a thermodynamically favored PrP^Sc^ conformation that is permissive for PrP expressed in a wide range of mammalian species [31]. Despite this permissibility, however, attempts to orally transmit BSE to pigs and chickens have failed [32], [33].

**Figure 6:**
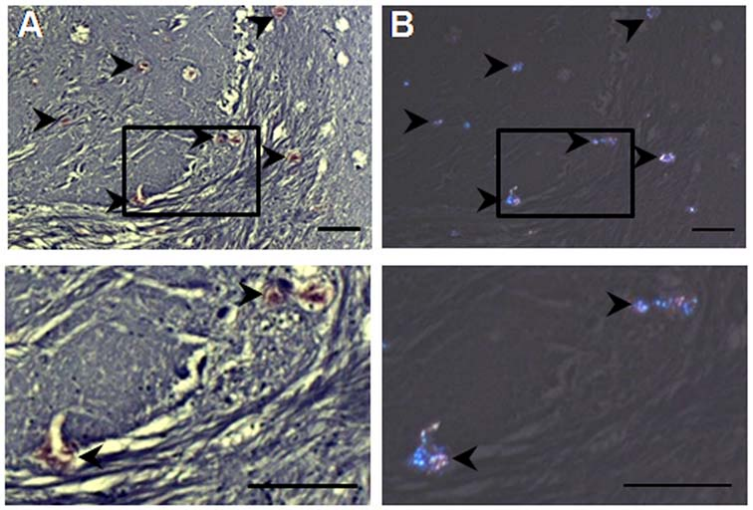
Congo red staining of deposits in the brain of BSE-challenged sea bream. A sagittal, 10 µm-brain section from a BSE-challenged individual, 24 months p.i., was stained with Congo red. A, Diencephalon with light microscopy; B, Same region in polarized light. Rectangles indicate areas of magnification shown in the panel directly below. Arrowheads indicate the abnormal aggregates. Scale bars, 100 µm.

**Figure 7:**
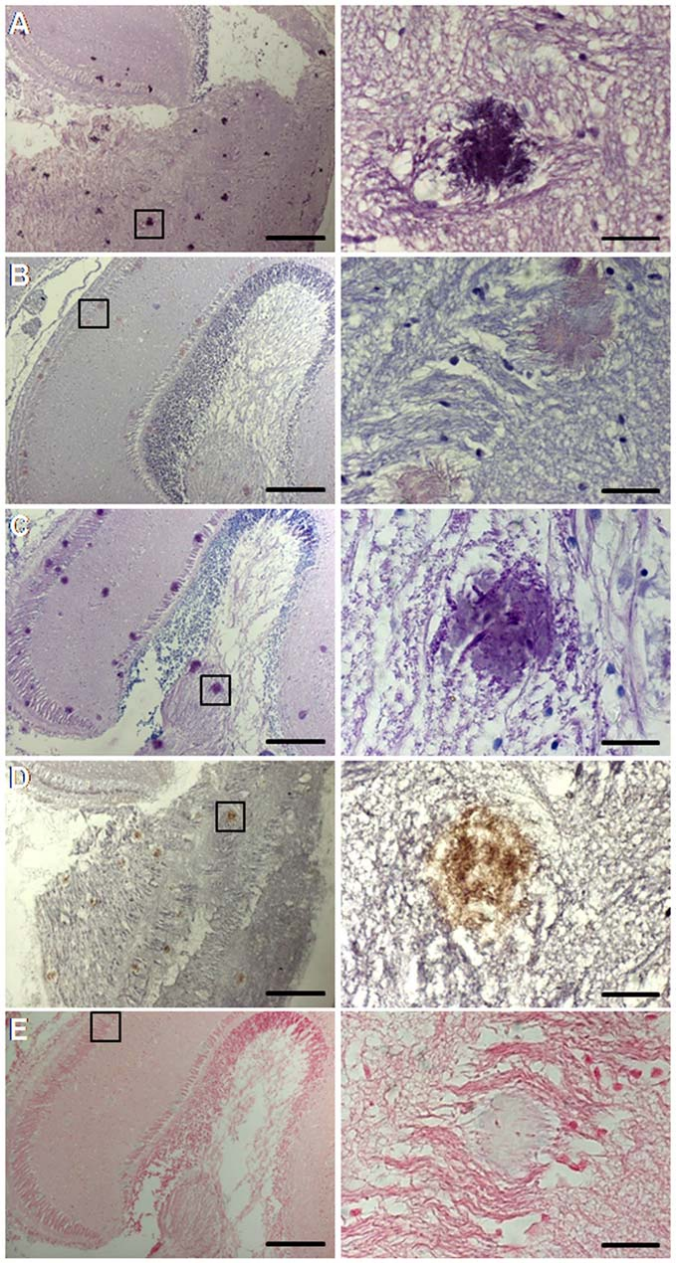
Staining of an aggregate in the brain of a BSE-challenged fish 24 months p.i. with different techniques. A, H&E; B, Congo red in normal light; C, PAS; D, IHC (SaurPrP1) after PK-digestion; E, Alcian blue. Rectangles in the left panel indicate areas of magnification shown in the right panel. Scale bars, 100 µm (left panel) or 10 µm (right panel).

To characterize the nature of the deposition, we employed a variety of conventional staining techniques. Congo red-stained deposits in BSE-challenged sea bream brains at 24 months p.i. were congophilic (Figs. 6A and 7B) and birefringent under polarized light (Fig. 6B), suggesting an amyloid or amyloid-like component [34]. No Congo red birefringence was observed in either the control tissues or the scrapie-challenged fish brains (data not shown). While the plaque-like aggregates were prominent with hematoxyline and eosin (H&E) (Fig. 7A), Klüver-Barrera staining for myelin structures and von Kossa staining for calcium deposition both gave negative reactions (data not shown). Deposits were also PAS positive (Fig. 7C) but Alcian blue negative (Fig. 7E), indicating the presence of carbohydrates and the absence of acidic glycosaminoglycans, respectively. Finally, our four anti-fish PrP antisera positively labeled the deposits (see Fig. 7D for SaurPrP1), whereas the 12F10 and 6H4 antibodies did not (data not shown).

**Figure 8:**
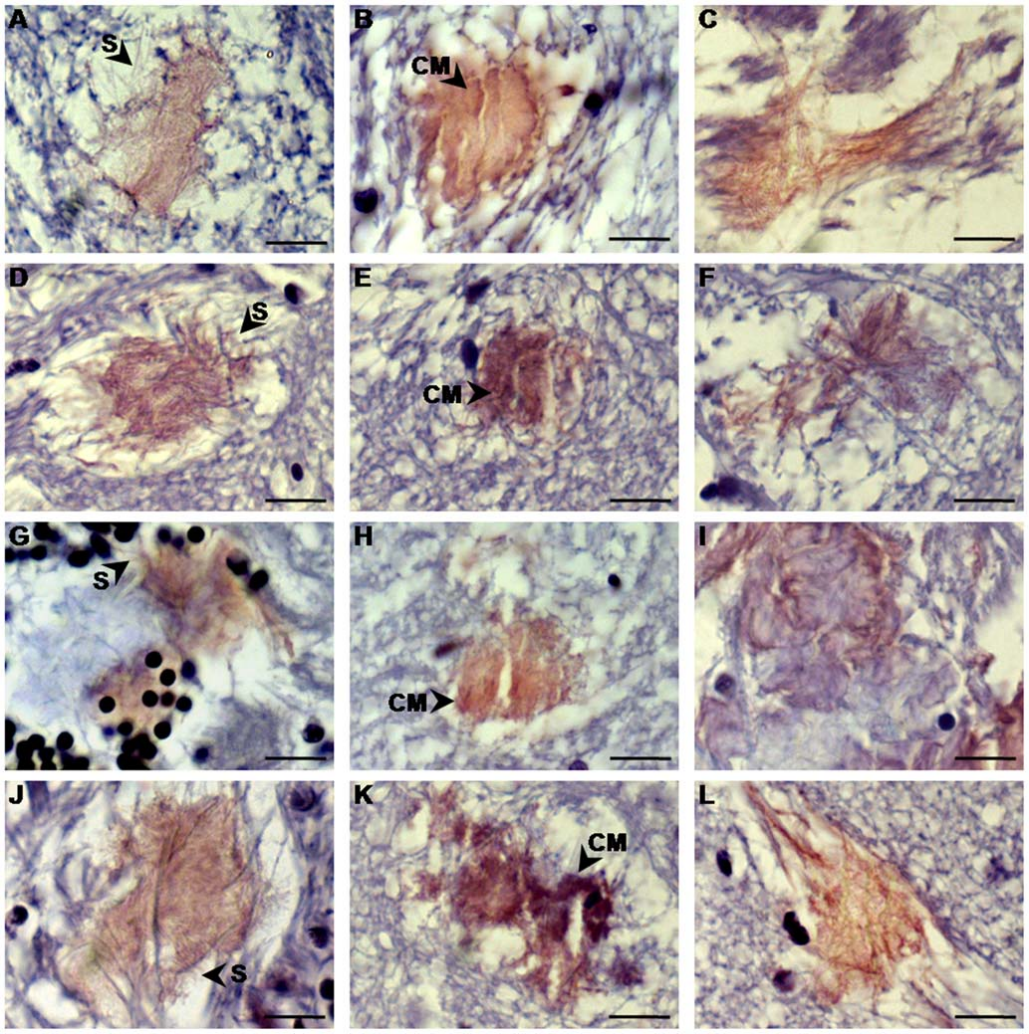
Morphology of the abnormal deposition in the brains of the BSE-challenged fish 24 months p.i.. Sagittal 4 µm-thick brain sections where stained with hematoxylin without a counterstain. pH variation renders the deposits visible by light microscopy. The panels on the left show diffuse “pre-mature” deposits in A, diencephalon; D, optic tectum; G, cerebellum; J, brainstem. The middle panels display examples of compact “mature” deposition in B, diencephalon; E, optic tectum; H, cerebellum; K, telencephalon. The panels at the right show intermediate stage-abnormal deposition within fiber bundles that disrupts synapses in C, diencephalon; F, optic tectum; I, valvula cerebelli; L, telencephalon. S, spicule-like projections; CM, condensed material. Scale bars, 10 µm.

Two main types of plaque-like deposits were identified in the brains of the BSE-challenged fish: fibrous, diffusely stained aggregates (Fig. 8A, D, G, J), and those that were more amorphous and dense (Fig. 8B, E, H, K). At 8 months, small aggregates, generally in close proximity to neurofibrils, were detected, whereas the majority of the adjacent fiber bundles remained intact (Fig. 3D–F). At 10 and 12 months the first signs of neurodegeneration appeared as a primitive disorganization of dendrites and axons. By 16 and 18 months, the distention of neurites, mostly in grey matter, was exacerbated. The extensive deconstruction of microfilaments within the axons and the loss of their coherence, especially at 18 months, were detected histopathologically. Aggregates of dystrophic neurites were immunostained with SaurPrP1, exhibiting a diffuse PrP-immunolabeling with some marginal spicule-like projections (data not shown). At 24 months the diffusely immunolabeled aggregates of dystrophic neurites (Fig. 8A, D, G, J) coexisted with deposits that appeared more amorphous, condensed and flocculated and therefore were more intensely stained with all the techniques used (Fig. 8B, E, H, K).

Given the morphological progression of the abnormal deposition with time, it is tempting to hypothesize a scenario, in which the first type of aggregates (Fig. 8A, D, G, J) could have been the developmental ancestor of the second (Fig. 8B, E, H, K) and in which each may illustrate different stages of pathogenesis. The distention of axons and dendrites observed at 10 and 12 months p.i. reflects an initial neurodegenerative process in the brains of the BSE-challenged fish that may have been a very early reaction following exposure to the infectious agent. The complete destruction of the protective outer neurite layers, including the myelin sheath, followed by the disorganization of the microfilaments and microtubuli could have subsequently created the first morphological type of aggregates initially detected at 16 and 18 months. These “pre-mature” deposits mainly consist of dystrophic neurites that have lost their coherence, with spicule-like projections, at their periphery (Fig. 8A, D, G, J). Given that aggregates were partially PK-resistant and showed affinity for Congo red by 24 months, the next step in the progression may have been the complete deconstruction of the fibers leading to the creation of a homogenous, flocculated, extracellular material that we describe as “mature” deposits (Fig. 8B, E, H, K), with increased PrP-immunopositivity, PK-resistance, congophilia and birefringence in polarized light [35]. Fish brains at 24 months post inoculation exhibit both types of abnormal aggregates, including intermediate states (Fig. 8C, F, I, L) that cannot be easily classified into any of the previously described morphological categories.

The classical neuropathological hallmarks of prion diseases include neurodegeneration, spongiform change and gliosis, while PrP^Sc^ deposition is observed in the majority of TSEs. The lesion profile in the brains of the BSE-challenged fish shares both similarities and differences in comparison to this histological and immunohistochemical pattern of mammalian prion diseases. Specifically, the distribution of abnormal aggregates within the brains of 4 BSE-challenged fish at 24 months p.i. shared certain similarities with the PrP^Sc^ deposition pattern observed in TSE-affected mammalian brain. Notably, the abnormal deposits in sea bream brain were only detected in regions where neuronal parenchyma was present, a feature greatly resembling the location of mammalian prion deposition [36]. Cerebellum was extensively affected, with large fibrous aggregates in the molecular layer and a granule-like deposition profile in the granular layer, a pattern similar to that observed in mammalian TSEs [37]. The abnormal deposition in sea bream brain was also prominent both in the lateral nucleus of the ventral telencephalic area, a fish counterpart to the basal nucleus of Meynert in mammals [38], and the lateral telencephalic pallium, homologue to the mammalian hippocampus [22]. Both thalamus and diencephalon displayed numerous aggregates, most of which were interspersed within neuronal fibers. In striking contrast to the general neuropathological profile of mammalian TSEs, however, no vacuoles were observed in any regions of the fish brains examined. While spongiosis is a main characteristic in most prion diseases, it must be noted that in certain TSE subtypes there is little or no spongiform change. Such has been the case in patients suffering from FFI, an inherited human prion disease [39].

Evidence of neurodegeneration, although distinct from that commonly associated with mammalian prion disease, was apparent in many brain regions, primarily in places where the abnormal deposition was located adjacent to or within complexes of neurites (Fig. 8C, L). It is important to note, however, that no degeneration associated with neuronal somata was detected in any of the anatomical regions examined. The absence of classically defined neurodegeneration might be related to the ability of fish to produce new neurons continuously throughout their lifetime. It is known, for instance, that adult fish can regenerate damaged retinal tissue, optic axons and descending brainstem axons, leading to functional recovery [40], [41]. In fact, this ability of adult fish for CNS regeneration has been postulated to explain the asymptomatic carrier state of halibut persistently infected with nodavirus [42]. In the present study it may have contributed to the “eclipse-like” temporal appearance of the abnormal deposition, as well as to the lack of clinical symptoms in our BSE-challenged fish.

Despite the positive IHC results, western blotting failed to detect PK-resistant PrP isoforms in the TSE-challenged fish brains (Fig. S5), possibly because the whole brain homogenates used did not have a high enough concentration of PK-resistant PrP to allow detection. In fact, it is clear from the IHC results that even at 24 months p.i., the brain regions associated with the abnormal deposits constitute only a small percentage of the whole brain mass.

The results of this TSE transmission study with gilthead sea bream indicate the development of a CNS histopathology in the brains of the fish challenged with the TSE-inocula. This neuropathology displays characteristics resembling a novel fish amyloidosis more than a classical TSE. Specifically, while the fish in our study showed no brain spongiosis and no clinical abnormalities, we did find numerous plaque-like deposits in the brains of a significant proportion of the BSE-challenged fish, especially. Although much of the PrP associated with these deposits is PK-sensitive, this should not be taken as an indicator of low potential infectivity, as instances of clinical prion disease, and even infectivity, associated with extremely low levels of detectable PK-resistant PrP have been reported [43]–[45].

In light of the serious ramifications that would follow an unequivocal demonstration of prion disease transmission to fish, it must be emphasized here that the abnormal deposition we observed in the brains of the TSE-challenged fish could possibly have resulted from pathogenic factors other than the prions they were fed. Despite the fact that no such naturally occurring, cross-species infections from mammals to fish have ever been reported [46], we cannot completely rule out this possibility. Thus, however unlikely, one must consider the possibility that the brains used to prepare the inocula for the TSE challenge were infected with an undetected virus or bacteria in addition to the scrapie or BSE present. Together, the time course of brain lesion appearance, i.e. months not days, the ability of the agent to survive the oral challenge route, the absence of brain histopathology in any of the control groups and the production of novel histological lesions in both the BSE- and the scrapie-challenged fish, in the absence of inflammation, however, make this possibility a remote one. A more plausible alternate explanation would be that the amyloidogenic nature of the TSE-inocula might have contributed to the development of a novel fish brain amyloidosis.

Infectivity and transmissibility are crucial issues that still need to be addressed. From a public health standpoint, the transmissibility of each prion strain and the relative ease with which it crosses species barriers, are its most significant characteristics. The spectrum of prionopathies, which has broadened in recent years, includes prion diseases that are not readily transmissible (*e.g*. some GSS cases), prion strains often associated with negligible clinical symptoms (*e.g*. the Nor98 scrapie strain), and even some without detectable PrP^Sc^ (*e.g*. PSPr) [44], [47], [48]. It is clear, then, that the evaluation and identification of both unusual prion diseases and prion diseases affecting unusual hosts is a complex task, requiring lengthy studies of pathogenesis, infectivity and transmissibility [49]. Until ongoing transmission studies using “bovinized” transgenic mice are completed, the possibility that the affected sea bream brain tissue might be infectious, must be taken seriously in any consideration to lift EU feed bans, especially those related to farmed fish.

## Materials and Methods

### Ethics Statement

All fish used in the experiments described in this work were treated in accordance with EU Council Directive 86/609/EEC for the protection of animals used for experimental and other scientific purposes.

### Fish

Sixteen hundred gilthead sea bream of approximately 20 g weight were purchased from a commercial farm (Interfish, Greece). At the commercial farm, before purchase, fish were fed commercial pellets (Biomar), none of which contained protein sources derived from land animal products. After transportation to the laboratory (Fisheries Research Institute, Kavala, Greece), they were maintained at 18°C in temperature-controlled recirculating water tanks. After a two week adaptation period, the fish were divided into groups of 200 in separate tanks. The fish were allowed a further three weeks of acclimatization before experimental manipulations were initiated.

### Preparation of inocula

For the force feeding of the fish, 10% (w/v) brain homogenates from scrapie-infected sheep, healthy control sheep, BSE-infected cow and healthy control cow were prepared in PBS (pH 7.4). For the sheep brain homogenates both cerebellum and brainstem from two animals were used (kindly provided by Dr. P. Toumazos, Veterinary Services, Cypriot Ministry of Agriculture), while the bovine brain homogenates were each prepared from the brainstem of a single animal. The BSE sample (RBSE 21028), taken in 1991 from a female Fresian two months after disease onset, was kindly provided by Dr. Ian Dexter, Pathology Department, Veterinary Laboratories Agency, Weybridge, UK. As healthy herdmate tissue was not available, the healthy control bovine brainstem was taken from a local Greek cow in 2002. All brain samples were stored at −80°C prior to use.

### Challenge and maintenance

For inoculations, fish were removed from the tanks and mildly anaesthetized with 0.3% ethylene glycol monophenyl ether. Following anaesthesia, each fish was force-fed 100 µl brain homogenate. In total, 2 groups of 400 fish each, were each treated with scrapie-infected or control sheep brain homogenate and 2 groups of 200 fish each were each treated with BSE-infected or control bovine brain homogenate. For both the experimental and control groups, the force-feeding procedure was repeated fortnightly for a total of five treatments, so that the cumulative inoculum for each fish was 50 mg brain equivalents. Following the inoculation period all fish were kept on a maintenance diet with commercially available chow to prevent excessive growth and overcrowding during the multiyear study period. Data regarding maintenance of the fish, mortality due to technical and natural causes and sampling are shown in Table S2.

### Clinical examination

Clinical evaluation of the fish in each tank was made on a daily basis, checking especially for any behavioral or swimming abnormalities.

### Histopathological evaluation

Individuals from each group (5 TSE-treated and 5 controls) were sacrificed at regular selected time points post inoculation (3, 6, 8, 10, 12, 14, 16, 18, 24 months) and tissue samples, including brain, spleen and intestine, were taken. Tissues were fixed in buffered formalin (pH 7.4), embedded in paraffin wax and finally 4 µm-thick serial sections were subjected to conventional staining with a variety of staining techniques including H&E, PAS, Alcian blue, von Kossa and Klüver-Barrera. The resulting sections were examined histologically using light microscopy (Axioplan 2 Imaging System, Zeiss). Tissue pictures were taken using the Nikon Digital Sight DS-SMc visualizing system.

### Congo red staining

For the identification of possible amyloid-like structures, 10 µm-thick brain sections were deparaffinized, stained with 0.5% Congo red (Merck, Darmstadt, Germany) alcohol solution for 15 minutes, destained in 0.2% KOH, subsequently counterstained with Mayer's hematoxyline, and after a short dehydration, they were finally cleared in xylene. The stained sections were observed microscopically under both normal and polarized light (Axiolab Carl Zeiss, rotatable analyzer +/−5°, 6×25, rotatable compensator Lambda, +/−5°, 6×25).

### Generation of polyclonal antisera

The presumed mature sequences spanning residues 24–580 of zebrafish (*Danio rerio*) PrP-1 and residues 18–539 of zebrafish PrP-2 (sequence data provided by Dr. Edward Málaga-Trillo, Department of Biology, University of Konstanz), were each amplified from genomic DNA, whereas the mature sequence of gilthead sea bream (*Sparus aurata*) PrP-1 spanning residues 26–475 was cloned from plasmid DNA. All three were cloned into the pET21d DNA vector (Novagen, San Diego, CA) to produce recombinant proteins tagged with six histidine residues at the C-terminus. After sequence verification by double-stranded sequencing, the recombinant proteins were expressed in BL21 (DE3) *E.coli* (Stratagene, La Jolla, CA) with IPTG induction from single clone colonies. The recombinant proteins were purified under denaturing conditions from cell lysates on Ni-NTA agarose columns (Qiagen, Hilden, Germany) and then specifically eluted with imidazole. The polyclonal antisera ZebPrP1, ZebPrP2 and SaurPrP1 were raised against the purified zebrafish PrP-1, zebrafish PrP-2 and sea bream PrP-1 proteins respectively, by 3 successive subcutaneous inoculations of rabbits with 150 to 200 µg of recombinant protein at 4 weeks intervals. All pertinent sequence data are deposited in GenBank and the accession numbers are given at the end of the manuscript.

### Affinity purification of SaurPrP1 polyclonal antiserum

2 aliquots containing 150 µg of gilthead sea bream recombinant PrP-1 protein each, were loaded onto 10% polyacrylamide gels and after SDS-PAGE, they were each electrotransferred to a nitrocellulose or a PVDF membrane. After electrophoresis the two membranes were stained with amido black staining solution (0.1%) and the protein-containing membrane pieces were finally excised. After a blocking step in 50 mM Tris HCl [pH 7.4], 150 mM NaCl, 0.05% Tween 20 (TBST) containing 5% milk, for 1 hr at RT, each membrane piece was probed with blocking buffer containing 500 µl of SaurPrP1 antiserum, at 4°C overnight. Following several washes, the IgGs that specifically bound to PrP-1, were finally eluted from the membranes with 0.2 M Glycine.HCl [pH 2.5], for 5 min at 4°C. Each eluate was neutralized with 2 M Tris HCl [pH 9.0], and then dialyzed overnight at 4°C in 50 mM Tris HCl [pH 7.4], 150 mM NaCl (TBS), using 100 mm-Spectra/Por molecularporous membrane tubing (Spectrum Medical Industries, Los Angeles, USA). Following dialysis, the purified IgGs were saturated in buffer containing 20 mM Tris HCl [pH 8.4], 150 mM NaCl, 5 mM EDTA, 1% gelatin, 0.1% BSA.

### Depletion of recombinant gilthead sea bream PrP-1 –specific immunoglobulin fraction from SaurPrP1 polyclonal antiserum

SaurPrP1 polyclonal antibody was diluted in phosphate buffered saline (1∶2000), containing 5% normal goat serum, 2.5% BSA and 0.05% Tween 20. The antibody was incubated with 0.6 mM of recombinant gilthead sea bream PrP-1 protein at 4°C overnight. The depleted antiserum was briefly centrifuged before use in all negative immunohistochemistry control experiments.

### Immunohistochemistry

Four different polyclonal anti-PrP antibodies were used for the immunohistochemical detection of the endogenous PrP proteins of sea bream, namely ZebPrP1 (1∶1000), ZebPrP2 (1∶1000), SaurPrP1 (1∶2000) and FuguPrP1 (1∶500), the latter being raised by our group against PrP-1 protein of *Takifugu rubripes*. The commercially available monoclonal antibody, 12F10 (Cayman Chemical, Ann Arbor, MI), raised against amino acids 142–160 of human PrP, was used for the detection of residual mammalian PrP (1∶200), since it also displays cross-reactivity with both ovine and bovine PrP. All paraffin sections were cut at 4 µm thickness. Depending on the prion protein of interest, PrP^C^ or PrP^Sc^, two different pretreatment protocols were used. For PrP^C^ labeling, an antigen retrieval step was performed by boiling in citrate-buffered saline [pH 6.0] for 7 minutes before the staining procedure. For PrP^Sc^ detection, the sections were hydrated-autoclaved at 121°C for 30 minutes, then incubated for 5 minutes in 90% formic acid prior to an 8 minutes-incubation with proteinase K (Dako, Glostrup, Denmark) at RT. Sections were treated with appropriate biotinylated secondary antibodies (Vector Laboratories, Burlingame, CA) and visualized using the avidin-biotin method-based Vectastain Elite ABC and the Diaminobezidine substrate kits (Vector Laboratories, Burlingame, CA) according to the manufacturer's instructions. Negative controls for immunohistochemistry involved omitting the primary antibody. Staining with polyclonal (anti 14-3-3β, Santa Cruz, California, USA) and monoclonal antibodies (12F10, 6H4) raised against proteins of mammalian origin was also performed. The mouse anti-tubulin monoclonal antibody (Abcam, Cambridge, UK) and the monoclonal SAF84 antibody (raised against SAF preparation from infected hamster brain, assumed epitope 126–164) were used as well.

### PrP^Sc^ enrichment and Western blot analysis

Western blot analysis of potentially enriched mammalian and teleost PrP^Sc^ was performed on brain homogenates from BSE-infected cows, scrapie-infected sheep, and TSE-challenged fish. Briefly, aliquots of 10% (w/v) brain homogenate were digested for 1 hr at 37°C with proteinase K at 25 µg/ml for sheep, 30 µg/ml for cow and 0.1–10 µg/ml for sea bream. PMSF (5 mM) was added to stop the protease digestion and PrP^Sc^ was precipitated with NaCl (10%) (w/v). The pellet was washed with 25 mM Tris HCl [pH 8.8] containing 0.05% sarkosyl and then resuspended in an appropriate volume of 2.5× O'Farrell buffer for gel electrophoresis.

For western blot analysis, untreated and proteinase K treated brain homogenates were analyzed by SDS-PAGE on 12% polyacrylamide gels and the separated proteins were then transferred onto PVDF membranes. After blocking with phosphate buffered saline containing 0.1% Tween 20 (PBST) and 5% milk, the immunoblots were probed with the fish-PrP specific polyclonal antisera, ZebPrP1 (1∶10000), ZebPrP2 (1∶35000), SaurPrP1 (1∶20000), FuguPrP1 (1∶20000), and the monoclonal antibody 6H4 (1∶5000) (Prionics, Zurich, Switzerland) overnight at 4°C. After washing, they were incubated for 1 hr with either alkaline-phosphatase or horseradish-peroxidase conjugated secondary goat anti-rabbit or anti-mouse antibodies (Pierce, Rockford, IL) diluted 1∶10000 in PBST. The blots were developed using the CDP-Star chemiluminescent substrate (NE Biolabs, Beverly, MA), or the ECL Western blotting Substrate (Pierce, Rockford, IL), depending on the secondary antibody and according to the manufacturer's instructions.

### GenBank Accession Numbers

Danio rerio prion protein 1 coding sequuence: AY438683

Danio rerio prion protein 1: AAS00159

Danio rerio prion protein 2 coding sequence: AY438684

Danio rerio prion protein 2: AAS00160

Sparus aurata prion protein 1 coding sequence: ABB90540

## Supporting Information

Table S1 Percentage amino acid sequence homology between prion proteins of different species. Full sequences were aligned. The NCBI accession numbers of sequence data are: Homo sapien (human), AAC78725; Bos Taurus (cow), AAD19998; Ovis aries (sheep), CAE00188; Mus musculus (mouse), AAH06703; Mesocricetus auratus (hamster), AAA37092; Takifugu rubripes (fugu), AAN38988; Danio rerio (zebrafish) prion protein 1, AAS00159; Danio rerio prion protein 2, AAS00160; Sparus aurata (gilthead sea bream) prion protein 1, ABB90540. Sequence alignments were performed by ALIGN (version 2, Myers and Miller, CABIOS (1989) 4:11–17).
(0.09 MB TIF)

Table S2 Cumulative record of the number of fish maintained post challenge. A, Fish inoculated with either scrapie- or normal ovine brain homogenate. B, Fish inoculated with either BSE- or normal bovine brain homogenate.
(0.04 MB DOC)

Table S3 Cumulative record of brain tissue samples examined. A, BSE-challenged and bovine control fish samples. B, Srapie-challenged and ovine control fish samples.
(0.14 MB DOC)

Table S4 Cytoanatomical analysis of brains from BSE-challenged fish sacrificed 24 months post challenge. The deposits have been classified into 2 morphological categories. F, fibrillary, >10 µm in diameter; NF, non fibrillary, circular<10 µm in diameter. Plus and minus symbols indicate the abundance of deposits: −, 0; +, 1–5; ++, 6–15; +++, 16–50, ++++, >50. NP, anatomical region not present in section; Mol L, molecular layer; Gran L, granular layer; WM, white matter; Cx, cortex; Ce, cerebellum; Vc, valvula cerebelli; Tel, telencephalon; Di, diencephalon; OT, optic tectum; Br. st., brain stem.
(0.13 MB TIF)

Figure S1 Comparison of antibody specificities for the PrPs of gilthead sea bream, cow and sheep by western blot analysis. A, Five 0.4 mg brain equivalent-aliquots of gilthead sea bream brain homogenate were loaded onto a 12% SDS-PAGE gel. B, Alternating lanes of a 12% SDS-PAGE gel were loaded with 3 mg tissue equivalents of PrPSc-enriched (see Materials and Methods) bovine BSE brain homogenate (lanes 1, 3, 5, 7 & 9) and ovine scrapie brain homogenate (lanes 2, 4, 6, 8 & 10). The electrophoretically separated proteins were transferred to PVDF membranes that were cut into four sections (in B, each section included two adjacent lanes). Each section was stained with one of five primary antibodies: 6H4 (1∶5000; lanes A1, B1, B2); FuguPrP1 (1∶20000; lanes A2, B3, B4); ZebPrP1 (1∶10000; lanes A3, B5, B6); ZebPrP2 (1∶35000; lanes A4, B7, B8); SaurPrP1 (1∶20000; lanes A5, B9, B10). After incubation with the appropriate alkaline-phosphatase-conjugated secondary antibody, the blots were developed with the CDP-Star reagent. The arrow heads indicate the positions of the molecular mass markers: A, 62 kDa and 47.5 kDa; B, 32.5 kDa.
(0.28 MB TIF)

Figure S2 Antibody specificity in IHC. Sagittal, 4 µm-thick serial brain sections from control gilthead sea bream were treated immunohistochemically with four different primary antibodies, without proteinase K digestion. A, SaurPrP1 (1∶2000); B, ZebPrP2 (1∶2000); C, Pre-immune serum from the rabbit in which SaurPrP1 was raised (1∶2000); D, PrP-specific immunoglobulin-depleted SaurPrP1 (1∶2000). Arrowheads indicate the existence (A, B) or absence (C, D) of PrP-immunopositivity. SGP, striatum griseum periventriculare; SFP, striatum fibrosum profundum; SGC, striatum griseum centrale; SPFE, striatum plexiforme et fibrosum externum; SFM, striatum fibrosum marginale; ML, molecular layer of the valvula cerebelli; GL, granular layer of the valvula cerebelli. Scale bars, 100 µm.
(8.23 MB TIF)

Figure S3 Temporal observation of the brains from control fish challenged with normal ovine brain homogenate. Sagittal brain sections taken at 12 and 24 months p.i. from fish challenged with normal ovine brain homogenate were stained with H&E (A, D), or immunolabeled with SaurPrP1 (1∶2000) without PK-digestion (B, E) and with PK-digestion (C, F). Images show diencephalon. The mean number of deposits (per section of fish containing deposits) observed in different brain regions without PK-treatment is indicated by the fill-type in the schematic drawings at the far left. Abbreviations as in Figure 2 of the main manuscript. The following areas were not examined: OB; OIN; OC; P. Rectangles indicate areas of magnification shown in the panels directly below. Scale bars, 100 µm.
(3.00 MB TIF)

Figure S4 Temporal observation of the brains from control fish challenged with normal bovine brain homogenate. Sagittal brain sections taken at the indicated timepoints p.i. from fish challenged with normal bovine brain homogenate were stained with H&E (A, D, G), or immunolabeled with SaurPrP1 (1∶2000) without PK-digestion (B, E, H) and with PK-digestion (C, F, I). Images show diencephalon. The mean number of deposits (per section of fish containing deposits) observed in different brain regions without PK-treatment is indicated by the fill-type in the schematic drawings at the far left. Abbreviations as in Figure 2 of the main manuscript. The following areas were not examined: OB; OIN; OC; P. Rectangles indicate areas of magnification shown in the panels directly below. Scale bars, 100 µm.
(4.48 MB TIF)

Figure S5 Sensitivity to proteinase K treatment of TSE-challenged sea bream brain tissues 24 months p.i.. After a short purification treatment, 0.4 mg brain equivalents from brain homogenates of either scrapie- (lanes 1, 3, 5 & 7) or BSE-challenged (lanes 2, 4, 6 & 8) fish, were digested with increasing proteinase K concentrations (0 µg/ml, lanes 1 & 2; 0.1 µg/ml, lanes 3 & 4; 1 µg/ml, lanes 5 & 6; 10 µg/ml, lanes 7 & 8) for 1 hr at 37°C. The samples were analyzed on a 12% SDS-PAGE gel, then electrotransferred onto a PVDF membrane and probed with SaurPrP1 polyclonal antibody (1∶20000). After incubation with the appropriate secondary antibody, the immunoblots were finally developed with the ECL western blotting substrate. Arrowhead, 47.5 kDa.
(0.27 MB TIF)

Text S1 Statistical analysis of data derived from the scrapie - challenged group.
(0.02 MB DOC)
