# Supplementary material for: Evaluation of the Possible Transmission of BSE and Scrapie to Gilthead Sea Bream (Sparus aurata)
Source: PLoS One. 2009 Jul 28;4(7):e6175. doi: 10.1371/journal.pone.0006175 (PMC2712096; doi:10.1371/journal.pone.0006175)
Supplement: Table S2 — Cumulative record of the number of fish maintained post challenge. A, Fish inoculated with either scrapie- or normal ovine brain homogenate. B, Fish inoculated with either BSE- or normal bovine brain homogenate. (0.04 MB DOC) [file pone.0006175.s002.doc]

**A. Fish inoculated with either scrapie - (challenged) or normal ovine brain homogenate (controls)**

| **Fish subgroups** | **Number of individuals** |
| --- | --- |
| Initially challenged fish | 400 |
| Fish found dead in tank  (~4 fish/month)* | ~280 |
|
| Fish sacrificed for assays  (5 fish for IHC and Histopathology/time point & 5 fish for Western blot and transmission studies/time point)^(1)^ | 120 |
|

^(1)^Sampling time points post inoculation for both groups included:

1, 3, 5, 6, 7, 8, 10, 12, 14, 16, 18 and 24 months. Only fish sacrificed at selected time points were used for immunohistochemical and histopathological analysis as described in the Materials and Methods section.

*At 6 months post inoculation half of the fish (~180) from each group died during a power failure that interrupted aeration in the tanks.

**B. Fish inoculated with either BSE - (challenged) or normal bovine brain homogenate (controls)**

| **Fish subgroups** | **Number of individuals** |
| --- | --- |
| Initially challenged fish | 200 |
| Fish found dead in tank  (~4 fish/month) | 100 |
|
| Fish sacrificed for assays  (5 fish for IHC and Histopathology/time point & 5 fish for Western blot and transmission studies/time point) ^(2)^ | 100 |
|

^(2)^Sampling time points post inoculation for both groups included: 3, 5, 6, 8, 10, 12, 14, 16, 18 and 24 months. Only fish sacrificed at selected time points were used for immunohistochemical and histopathological analysis.
