# Supplementary material for: Evaluation of the Possible Transmission of BSE and Scrapie to Gilthead Sea Bream (Sparus aurata)
Source: PLoS One. 2009 Jul 28;4(7):e6175. doi: 10.1371/journal.pone.0006175 (PMC2712096; doi:10.1371/journal.pone.0006175)
Supplement: Table S3 — Cumulative record of brain tissue samples examined. A, BSE-challenged and bovine control fish samples. B, Srapie-challenged and ovine control fish samples. (0.14 MB DOC) [file pone.0006175.s003.doc]

**A. BSE – challenged fish and controls**

| 8 months |  | Sections examined | Total aggregates per section | PK-resistant aggregates per section | PK-resistance (%) |
| --- | --- | --- | --- | --- | --- |
| Control fish | Individual 1 | 4 | 0 | 0 | 0 |
|  | Individual 2 | 4 | 0 | 0 | 0 |
|  | Individual 3 | 4 | 0 | 0 | 0 |
|  | Individual 4 | 4 | 0 | 0 | 0 |
|  | Individual 5 | 4 | 0 | 0 | 0 |
| BSE-challenged fish | Individual 1 | 4 | 235 | 18 | 7.6 |
|  | Individual 2 | 4 | 40 | 5 | 12.5 |
|  | Individual 3 | 4 | 50 | 6 | 12.0 |
|  | Individual 4 | 4 | 180 | 12 | 6.6 |
|  | Individual 5 | 4 | 130 | 10 | 7.7 |

| 10 months |  | Sections examined | Total aggregates per section | PK-resistant aggregates per section | PK-resistance (%) |
| --- | --- | --- | --- | --- | --- |
| Control fish | Individual 1 | 4 | 0 | 0 | 0 |
|  | Individual 2 | 4 | 0 | 0 | 0 |
|  | Individual 3 | 4 | 0 | 0 | 0 |
|  | Individual 4 | 4 | 0 | 0 | 0 |
|  | Individual 5 | 4 | 0 | 0 | 0 |
| BSE-challenged fish | Individual 1 | 4 | 40 | 6 | 15.0 |
|  | Individual 2 | 4 | 0 | 0 | 0 |
|  | Individual 3 | 4 | 0 | 0 | 0 |
|  | Individual 4 | 4 | 165 | 35 | 21.2 |
|  | Individual 5 | 4 | 185 | 25 | 13.5 |

| 12 months |  | Sections examined | Total aggregates per section | PK-resistant aggregates per section | PK-resistance (%) |
| --- | --- | --- | --- | --- | --- |
| Control fish | Individual 1 | 8 | 0 | 0 | 0 |
|  | Individual 2 | 8 | 0 | 0 | 0 |
|  | Individual 3 | 8 | 0 | 0 | 0 |
|  | Individual 4 | 8 | 0 | 0 | 0 |
|  | Individual 5 | 8 | 0 | 0 | 0 |
| BSE-challenged fish | Individual 1 | 8 | 100 | 35 | 35.0 |
|  | Individual 2 | 8 | 135 | 65 | 48.1 |
|  | Individual 3 | 8 | 145 | 45 | 31.0 |
|  | Individual 4 | 8 | 195 | 135 | 69.2 |
|  | Individual 5 | 8 | 35 | 25 | 71.4 |

| 14 months |  | Sections examined | Total aggregates per section | PK-resistant aggregates per section | PK-resistance (%) |
| --- | --- | --- | --- | --- | --- |
| Control fish | Individual 1 | 8 | 0 | 0 | 0 |
|  | Individual 2 | 8 | 0 | 0 | 0 |
|  | Individual 3 | 8 | 0 | 0 | 0 |
|  | Individual 4 | 8 | 0 | 0 | 0 |
|  | Individual 5 | 8 | 0 | 0 | 0 |
| BSE-challenged fish | Individual 1 | 8 | 95 | 60 | 63.1 |
|  | Individual 2 | 8 | 0 | 0 | 0 |
|  | Individual 3 | 8 | 35 | 20 | 57.1 |
|  | Individual 4 | 8 | 35 | 15 | 42.8 |
|  | Individual 5 | 8 | 220 | 145 | 65.9 |

| 16 months |  | Sections examined | Total aggregates per section | PK-resistant aggregates per section | PK-resistance (%) |
| --- | --- | --- | --- | --- | --- |
| Control fish | Individual 1 | 8 | 0 | 0 | 0 |
|  | Individual 2 | 8 | 0 | 0 | 0 |
|  | Individual 3 | 8 | 0 | 0 | 0 |
|  | Individual 4 | 8 | 0 | 0 | 0 |
|  | Individual 5 | 8 | 0 | 0 | 0 |
| BSE-challenged fish | Individual 1 | 8 | 50 | 25 | 50.0 |
|  | Individual 2 | 8 | 80 | 55 | 68.7 |
|  | Individual 3 | 8 | 90 | 30 | 33.3 |
|  | Individual 4 | 8 | 0 | 0 | 0 |
|  | Individual 5 | 8 | 90 | 50 | 55.5 |

| 18 months |  | Sections examined | Total aggregates per section | PK-resistant aggregates per section | PK-resistance (%) |
| --- | --- | --- | --- | --- | --- |
| Control fish | Individual 1 | 10 | 0 | 0 | 0 |
|  | Individual 2 | 10 | 0 | 0 | 0 |
|  | Individual 3 | 10 | 0 | 0 | 0 |
|  | Individual 4 | 10 | 0 | 0 | 0 |
|  | Individual 5 | 10 | 0 | 0 | 0 |
| BSE-challenged fish | Individual 1 | 10 | 0 | 0 | 0 |
|  | Individual 2 | 10 | 50 | 35 | 70.0 |
|  | Individual 3 | 10 | 5 | 0 | 0 |
|  | Individual 4 | 10 | 77 | 55 | 71.4 |
|  | Individual 5 | 10 | 50 | 35 | 70.0 |

| 24 months |  | Sections examined | Total aggregates per section | PK-resistant aggregates per section | PK-resistance (%) |
| --- | --- | --- | --- | --- | --- |
| Control fish | Individual 1 | 30 | 0 | 0 | 0 |
|  | Individual 2 | 30 | 0 | 0 | 0 |
|  | Individual 3 | 30 | 0 | 0 | 0 |
|  | Individual 4 | 30 | 0 | 0 | 0 |
|  | Individual 5 | 30 | 0 | 0 | 0 |
| BSE-challenged fish | Individual 1 | 30 | 735 | 625 | 85.0 |
|  | Individual 2 | 30 | 500 | 360 | 72.0 |
|  | Individual 3 | 30 | 785 | 650 | 82.8 |
|  | Individual 4 | 30 | 5 | 0 | 0 |
|  | Individual 5 | 30 | 145 | 135 | 93.1 |

**B. Scrapie – challenged fish and controls**

| 24 months |  | Sections examined | Total aggregates per section | PK-resistant aggregates per section | PK-resistance (%) |
| --- | --- | --- | --- | --- | --- |
| Control fish | Individual 1 | 10 | 0 | 0 | 0 |
|  | Individual 2 | 10 | 0 | 0 | 0 |
|  | Individual 3 | 10 | 0 | 0 | 0 |
|  | Individual 4 | 10 | 0 | 0 | 0 |
|  | Individual 5 | 10 | 0 | 0 | 0 |
| Scrapie-challenged fish | Individual 1 | 10 | 35 | 0 | 0 |
|  | Individual 2 | 10 | 0 | 0 | 0 |
|  | Individual 3 | 10 | 0 | 0 | 0 |
|  | Individual 4 | 10 | 70 | 0 | 0 |
|  | Individual 5 | 10 | 0 | 0 | 0 |
