# Supplementary figures and images for: Evaluation of the Possible Transmission of BSE and Scrapie to Gilthead Sea Bream (Sparus aurata)
Source: PLoS One. 2009 Jul 28;4(7):e6175. doi: 10.1371/journal.pone.0006175 (PMC2712096; doi:10.1371/journal.pone.0006175)

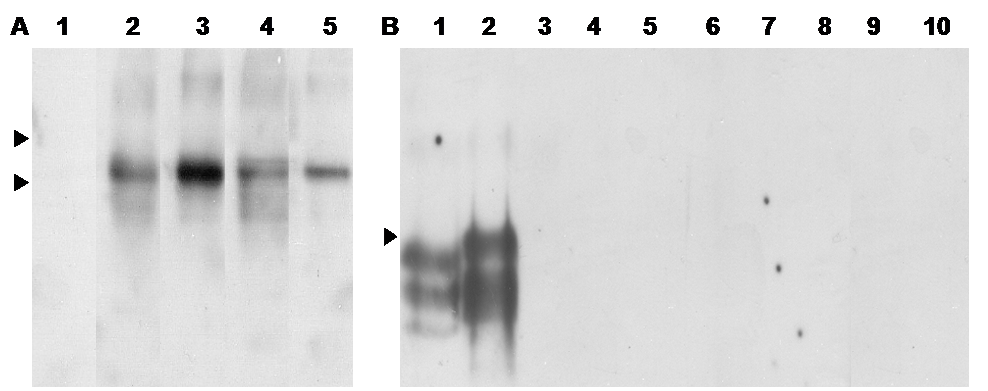

Supplement: Figure S1 — Comparison of antibody specificities for the PrPs of gilthead sea bream, cow and sheep by western blot analysis. A, Five 0.4 mg brain equivalent-aliquots of gilthead sea bream brain homogenate were loaded onto a 12% SDS-PAGE gel. B, Alternating lanes of a 12% SDS-PAGE gel were loaded with 3 mg tissue equivalents of PrPSc-enriched (see Materials and Methods) bovine BSE brain homogenate (lanes 1, 3, 5, 7 & 9) and ovine scrapie brain homogenate (lanes 2, 4, 6, 8 & 10). The electrophoretically separated proteins were transferred to PVDF membranes that were cut into four sections (in B, each section included two adjacent lanes). Each section was stained with one of five primary antibodies: 6H4 (1∶5000; lanes A1, B1, B2); FuguPrP1 (1∶20000; lanes A2, B3, B4); ZebPrP1 (1∶10000; lanes A3, B5, B6); ZebPrP2 (1∶35000; lanes A4, B7, B8); SaurPrP1 (1∶20000; lanes A5, B9, B10). After incubation with the appropriate alkaline-phosphatase-conjugated secondary antibody, the blots were developed with the CDP-Star reagent. The arrow heads indicate the positions of the molecular mass markers: A, 62 kDa and 47.5 kDa; B, 32.5 kDa. (0.28 MB TIF) [file pone.0006175.s005.tif]

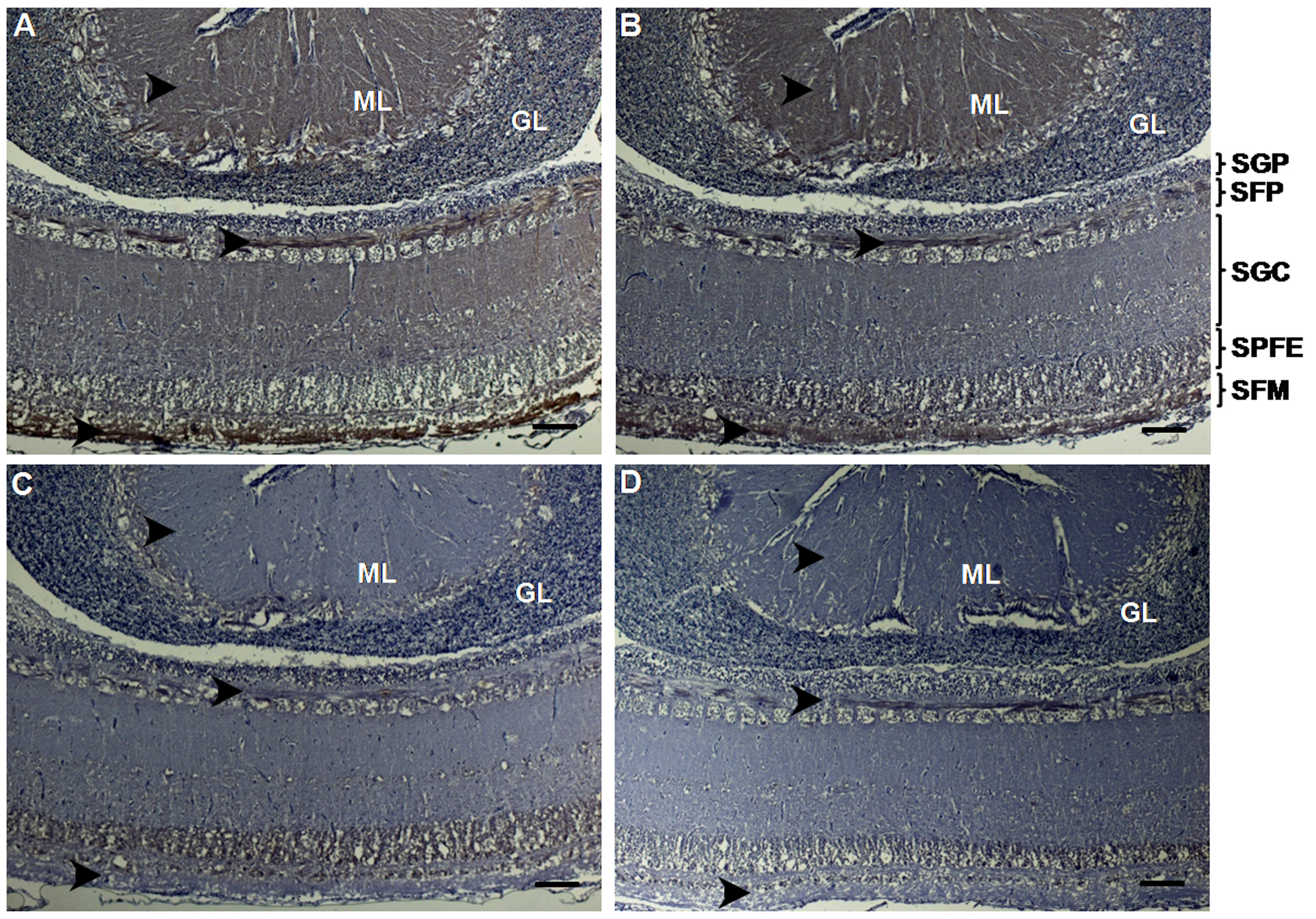

Supplement: Figure S2 — Antibody specificity in IHC. Sagittal, 4 µm-thick serial brain sections from control gilthead sea bream were treated immunohistochemically with four different primary antibodies, without proteinase K digestion. A, SaurPrP1 (1∶2000); B, ZebPrP2 (1∶2000); C, Pre-immune serum from the rabbit in which SaurPrP1 was raised (1∶2000); D, PrP-specific immunoglobulin-depleted SaurPrP1 (1∶2000). Arrowheads indicate the existence (A, B) or absence (C, D) of PrP-immunopositivity. SGP, striatum griseum periventriculare; SFP, striatum fibrosum profundum; SGC, striatum griseum centrale; SPFE, striatum plexiforme et fibrosum externum; SFM, striatum fibrosum marginale; ML, molecular layer of the valvula cerebelli; GL, granular layer of the valvula cerebelli. Scale bars, 100 µm. (8.23 MB TIF) [file pone.0006175.s006.tif]

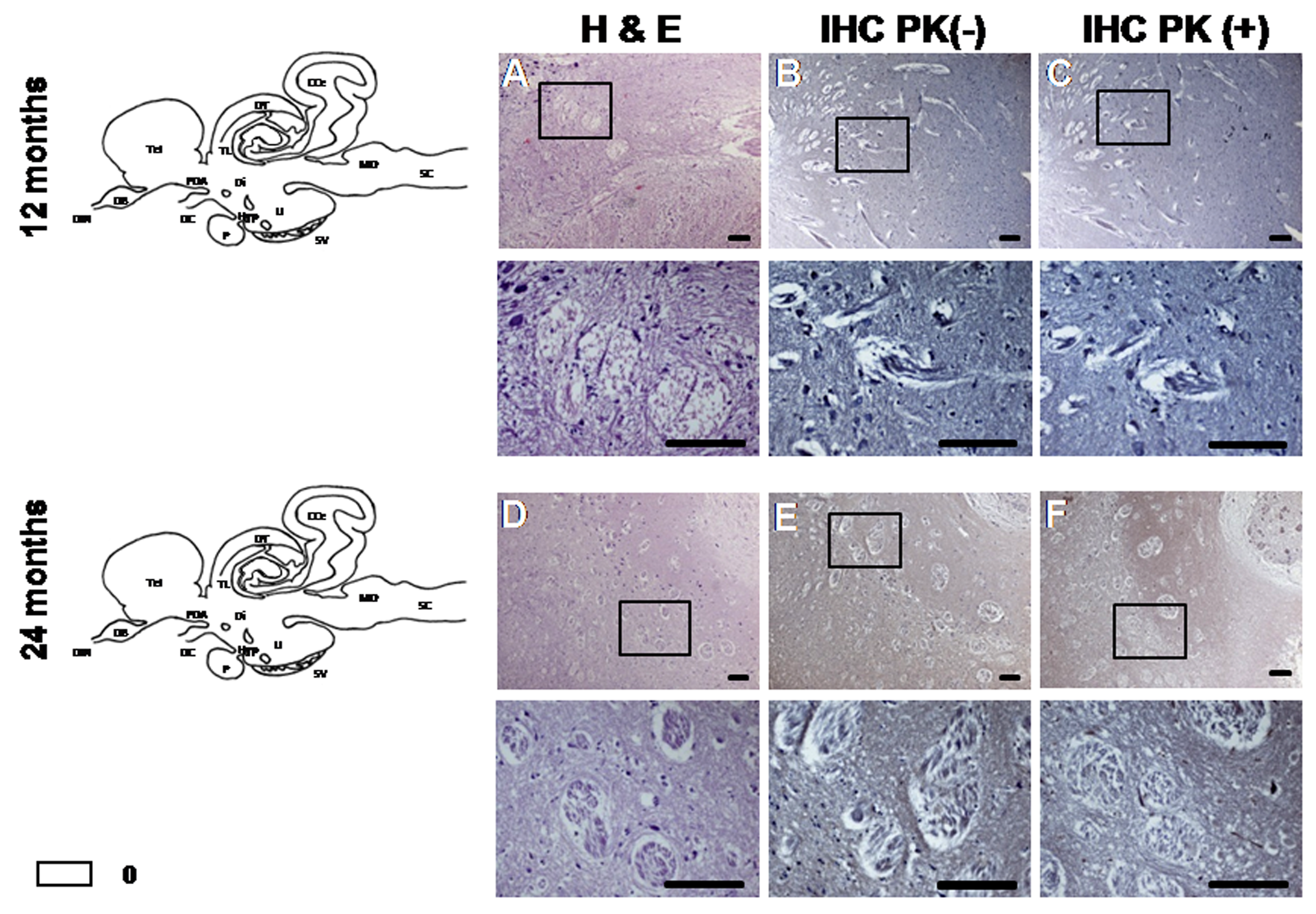

Supplement: Figure S3 — Temporal observation of the brains from control fish challenged with normal ovine brain homogenate. Sagittal brain sections taken at 12 and 24 months p.i. from fish challenged with normal ovine brain homogenate were stained with H&E (A, D), or immunolabeled with SaurPrP1 (1∶2000) without PK-digestion (B, E) and with PK-digestion (C, F). Images show diencephalon. The mean number of deposits (per section of fish containing deposits) observed in different brain regions without PK-treatment is indicated by the fill-type in the schematic drawings at the far left. Abbreviations as in Figure 2 of the main manuscript. The following areas were not examined: OB; OIN; OC; P. Rectangles indicate areas of magnification shown in the panels directly below. Scale bars, 100 µm. (3.00 MB TIF) [file pone.0006175.s007.tif]

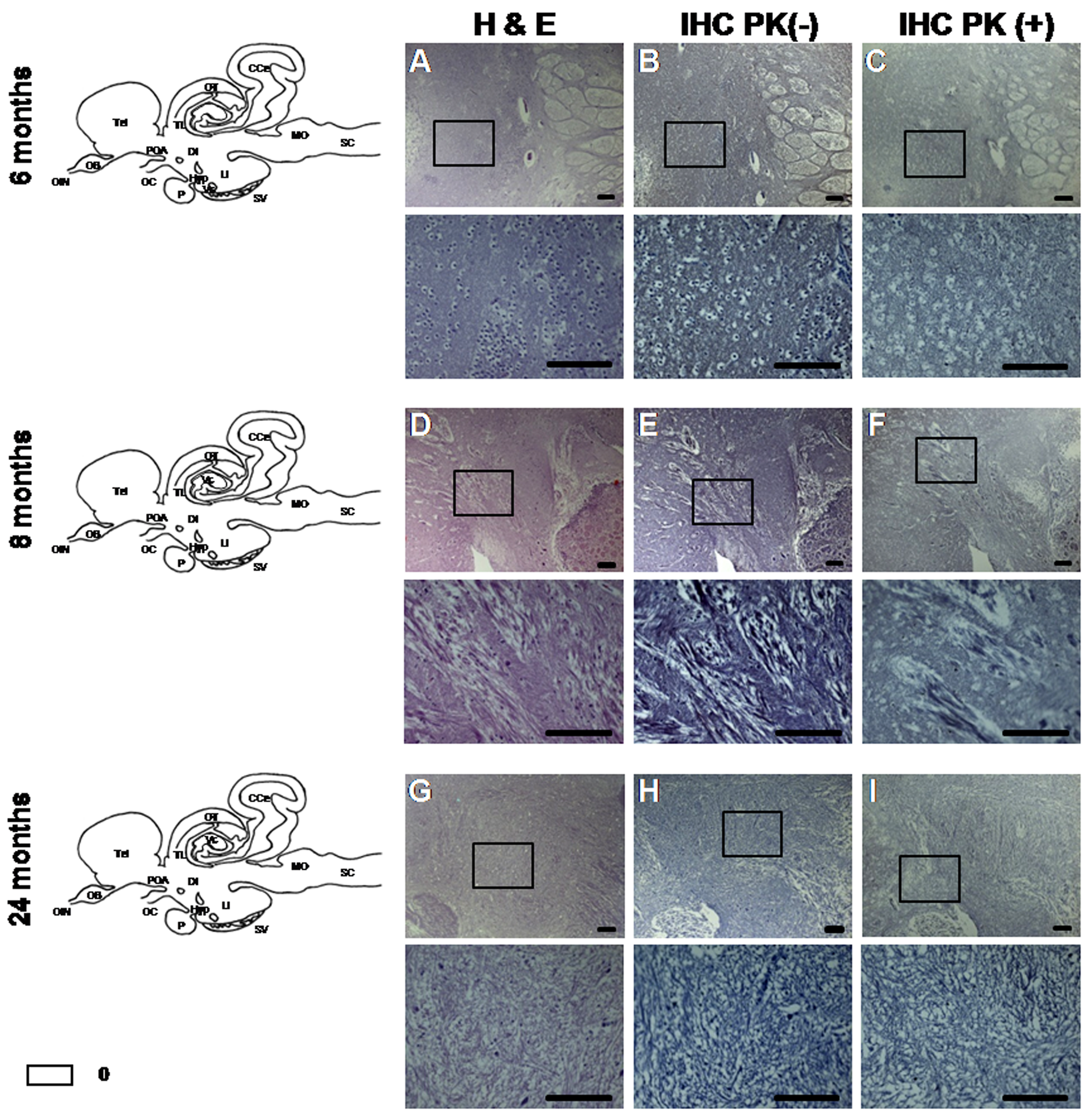

Supplement: Figure S4 — Temporal observation of the brains from control fish challenged with normal bovine brain homogenate. Sagittal brain sections taken at the indicated timepoints p.i. from fish challenged with normal bovine brain homogenate were stained with H&E (A, D, G), or immunolabeled with SaurPrP1 (1∶2000) without PK-digestion (B, E, H) and with PK-digestion (C, F, I). Images show diencephalon. The mean number of deposits (per section of fish containing deposits) observed in different brain regions without PK-treatment is indicated by the fill-type in the schematic drawings at the far left. Abbreviations as in Figure 2 of the main manuscript. The following areas were not examined: OB; OIN; OC; P. Rectangles indicate areas of magnification shown in the panels directly below. Scale bars, 100 µm. (4.48 MB TIF) [file pone.0006175.s008.tif]
