# Supplementary material for: Evaluation of the Possible Transmission of BSE and Scrapie to Gilthead Sea Bream (Sparus aurata)
Source: PLoS One. 2009 Jul 28;4(7):e6175. doi: 10.1371/journal.pone.0006175 (PMC2712096; doi:10.1371/journal.pone.0006175)
Supplement: Text S1 — Statistical analysis of data derived from the scrapie - challenged group. (0.02 MB DOC) [file pone.0006175.s010.doc]

When the response variable can be viewed as the number of “successes” out of a fixed number of trials, the standard distribution to use is the binomial. In the case that large frequencies of zeros are observed relative to what is predicted by the binomial distribution, we make the assumption that the data are generated by a mixture of a binomial with a degenerate distribution with point mass of 1 at zero. This is the zero inflated binomial model (ZIB). In our case the observations $Y_{i}$ i=1,…, N (N=9=No of timepoints checked) are counts which can be thought of as the number of “successes” occurring out of n=5 trials. We assume that a sample (within the scrapie-challenged group) is in the perfect state (no plaques observed) with probability p and the imperfect state (has developed plaques) with probability 1-p. If a sample is in the perfect state, $Y_{i}$ takes only the value zero. If the sample is in the imperfect state, the random variable $Y_{i}$ represents the number of successes in n=5 trials with probability of success in each trial π; that is it follows the binomial distribution $B(n_{i},\pi)$ and can take the values 0, 1… , 5.

We fitted a ZIB distribution to the data 0/5, 0/5, 0/5, 0/5, 0/5, 0/5, 0/5, 0/5, 2/5 using the function zibinomial {VGAM} implemented in R (R Development Core Team 2007, R: A language and Environment for Statistical Computing. URLhttp://www.R-project.org/). The maximum likelihood estimate of the parameter p, the probability of a sample belonging to the perfect state is 0.9062453 and the maximum likelihood of the binomial probability is 0.355544 .
